# The complete chloroplast genome of *Phyllostachys angusta* McClure (Poaceae)

**DOI:** 10.1080/23802359.2020.1860703

**Published:** 2021-01-16

**Authors:** Zheng Yu, Zhao Linying, Wang Tao, Chen Guofu, Hong Yifeng, Yue Jinjun

**Affiliations:** a East China Inventory and Planning Institute of National Forestry and Grassland Administration, Hangzhou, China; b Research Institute of Subtropical Forestry, Chinese Academy of Forestry, Hangzhou, China

**Keywords:** *Phyllostachys angusta* McClure, chloroplast genome, phylogenetic analysis, sequencing

## Abstract

*Phyllostachys angusta* McClure is a precious wood-use bamboo resource, with almost straight stem. The complete chloroplast genome of the *P. angusta* McClure was assembled for the first time from Illumina pair-end sequencing data in this work. The total genome size of *P. angusta* McClure was 139,678 bp in length, containing a large single-copy (LSC) region of 83,212 bp, a small single-copy (SSC) region of 12,870 bp, and a pair of inverted repeat (IR) regions of 21,798 bp. The overall GC content of the genome was 38.89%, and the corresponding values of the LSC, SSC, and IR regions were 36.97, 33.17, and 44.22%, respectively. A total of 131 genes were annotated, including 85 protein-coding genes, 36 tRNA genes, and 8 rRNA genes. Phylogenetic analysis results strongly supported that *P. angusta* McClure was closely related to *P. reticulate*.

The culm wall of *P. angusta* McClure, belonging to Phyllostachys, is particularly straight, which is mainly cultivated in Zhejiang Provinces of China. The chloroplast genomes of *Phyllostachys* genus have been reported as *Phyllostachys edulis* cultivar pachyloen, *Phyllostachys edulis*, *Phyllostachys nigra* var. *henonis*, *Phyllostachys reticulata*, and *Phyllostachys sulphurea* (Zhang et al. 2011; Wu and Ge 2012; Gao and Gao 2016; Huang et al. 2020). In the present study, we reported the complete cp genome sequence of *Phyllostachys angusta* McClure based on Illumina pair-end data for the first time. We also explored its phylogenetic relationship with other plant species, which would help our better understanding of the evolution of *Phyllostachys* cp genome.

The fresh leaves of *Phyllostachys angusta* McClure were collected from the experimental bamboo forest (119.236° E, 30.698° N, 36.9 m above sea level) in Shimen Town, Xuancheng County, Anhui Province, China. The voucher specimens have been deposited in East China Inventory and Planning Institute of National Forestry and Grassland Administration (20200813). Total genome DNA was extracted with the Qiagen plant genomic DNA prep kit (Sangon Biotech, Shanghai, China), which were sequence using the Illumina HiSeq 2500 platform. The library with insert size of 300 bp fragments was constructed and sequenced using the Illumina HiSeq platform in Novogene (Nanjing, China). The raw data were used to assemble the complete cp genome using Getorganelle software (Jin et al. 2018) with *Phyllostachys edulis* as the reference. Genome annotation was performed with the program Geneious R8 (Biomatters Ltd, Auckland, New Zealand) by comparing the sequences with the cp genome of *Phyllostachys edulis*, coupled with manual. The tRNA genes were further confirmed through online tRNAscan-SE web servers (Schattner et al. 2005). A gene map of the annotated *P. heteroclada* f. *solida* cp genome was drawn by OGdraw online (Lohse et al. 2013). Furthermore, the cp genome data of *Phyllostachys angusta* McClure was available in the NCBI under accession number of MW027348 (https://www.ncbi.nlm.nih.gov/nuccore/MW027348).

The cp genome of *Phyllostachys angusta* McClure was a quadripartite circular with 139,678 bp, which comprised of a large-single copy (LSC) region of 83,212 bp and a small single copy (SSC) region of 12,870 bp, separated by two inverted repeat (IR) regions of 21,798 bp, respectively. The GC content of the total genome was 36.12%, whereas the IR region had a higher GC content (44.22%) than LSC (36.97%) and SSC (33.17%). The cp genome encoded 131 genes, including 85 protein-coding genes, 36 tRNA genes, and 8 rRNA genes.

In order to study the relationship between *Phyllostachys angusta* McClure and other Phyllostachys plants, the cp genome data of six species of Phyllostachys (*Phyllostachys edulis* var. Pachyloen, *Phyllostachys edulis*, *Phyllostachys nigra* var. *henonis*, *Phyllostachys sulphurea*, *Phyllostachys propinqua* and *Phyllostachys reticulate*), five species of Arundinaria (*Arundinaria fargesii*, *Arundinaria humbertii*, *Arundinaria gigantean*, *Arundinaria appalachiana* and *Arundinaria tecta*) have been published in the NCBI gene library were used to align by MAFFT v7.313 (Katoh and Standley 2013) and construct phylogenetic trees (Figure 1). Phylogenetic analysis results strongly supported that *Phyllostachys angusta* McClure was closely related to *Phyllostachys reticulate*.

**Figure 1.**
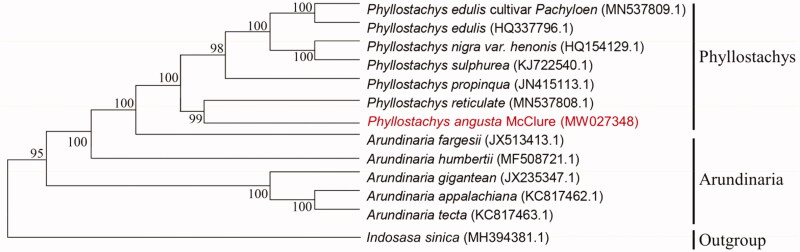
Phylogenetic relationships among 12 complete chloroplast genomes of *Phyllostachys* and *Arundinaria*. Bootstrap support values are given at the nodes.

## Data Availability

The genome sequence data that support the findings of this study are openly available in GenBank of NCBI at (https://www.ncbi.nlm.nih.gov/) under the accession no. MW027348. The associated BioProject, SRA, and Bio-Sample numbers are PRJNA642983, SRS6922745, and SAMN15402429, respectively.
